# HIV and Cocaine Impact Glial Metabolism: Energy Sensor AMP-activated protein kinase Role in Mitochondrial Biogenesis and Epigenetic Remodeling

**DOI:** 10.1038/srep31784

**Published:** 2016-08-18

**Authors:** Thangavel Samikkannu, Venkata S. R. Atluri, Madhavan P. N. Nair

**Affiliations:** 1 Department of Immunology, Institute of NeuroImmune Pharmacology, Herbert Wertheim College of Medicine, Florida International University, Miami, Florida 33199, USA.

## Abstract

HIV infection and cocaine use have been identified as risk factors for triggering neuronal dysfunction. In the central nervous system (CNS), energy resource and metabolic function are regulated by astroglia. Glia is the major reservoir of HIV infection and disease progression in CNS. However, the role of cocaine in accelerating HIV associated energy deficit and its impact on neuronal dysfunction has not been elucidated yet. The aim of this study is to elucidate the molecular mechanism of HIV associated neuropathogenesis in cocaine abuse and how it accelerates the energy sensor AMPKs and its subsequent effect on mitochondrial oxidative phosphorylation (OXPHOS), BRSKs, CDC25B/C, MAP/Tau, Wee1 and epigenetics remodeling complex SWI/SNF. Results showed that cocaine exposure during HIV infection significantly increased the level of p24, reactive oxygen species (ROS), ATP-utilization and upregulated energy sensor AMPKs, CDC25B/C, MAP/Tau and Wee1 protein expression. Increased ROS production subsequently inhibits OCR/ECAR ratio and OXPHOS, and eventually upregulate epigenetics remodeling complex SWI/SNF in CHME-5 cells. These results suggest that HIV infection induced energy deficit and metabolic dysfunction is accelerated by cocaine inducing energy sensor AMPKs, mitochondrial biogenesis and chromatin remodeling complex SWI/SNF activation, which may lead to neuroAIDS disease progression.

HIV infection is known to target microglia, and subsequently impact astrocytes and neurons in the central nervous system (CNS) dysfunction^1^,^2^. Illicit drugs including cocaine are significant risk factor for HIV infection and AIDS disease progression^3^,^4^,^5^. Cocaine abuse can lead to the development of neuropathogenesis by altering neurotransmitter systems in the brain^6^ and affecting glial function^7^,^8^,^9^. Glial cells (astrocytes, oligodendrocytes and microglia) play essential roles in the supply of energy metabolites for synaptic function, neuronal polarity, axons and dendritic formation^10^,^11^. HIV infection and cocaine use are known to affect neuronal function, which are mediated by glial cells^1^,^2^. Studies have shown that HIV-1 envelop glycoprotein 120 (gp120) is predominantly found in HIV positive postmortem brains and it is required for viral entry, facilitating the viral replication and disease progression in CNS^12^,^13^.

Energy source of intracellular ATP depletion known to threaten cellular homeostasis and integrity. Impaired energy metabolism may trigger pro-apoptotic signaling (programmed cell death), oxidative damage, excitotoxicity and impede mitochondrial damage^14^. Oxidative stress induced ROS production ultimately inhibits supply of energy sources and persuaded neuronal impairment is mediated by energy sensor 5′ adenosine monophosphate-activated protein kinase (AMPK)^15^. The AMPKs (an energy metabolism marker) is pivotal regulator of cellular function and it plays a wide role as a fuel sensor of cells or master switch of metabolic pathways^16^. AMPK is a heterotrimeric Ser/Thr protein kinase, consisting of a catalytic α subunit and two regulatory β and γ subunits^17^,^18^, It is predominantly expressed in most mammalian tissues and cell types, including CNS, where it is believed to play a critical role including energy consumption, glucose deprivation, and release of hormones and inflammatory cytokines^19^,^20^. Furthermore, the AMPK-related kinase family of total 12 kinases has been identified including brain-specific serine/threonine kinases (BRSK1), (BRSK2), nuclear AMPK-related kinases novel (nua) kinase family 1 (NUAK1), and sucrose non fermenting AMPK-related kinase (SNARK)/NUAK2. The BRSK1/2 (also named SAD-B/A) and SNARK proteins are closely associated with energy metabolism^21^. The BRSKs are highly expressed in mammalian forebrain, present in hippocampal neurons and play a role in neurotransmission, and synaptic vesicle distribution, and development of normal synapses^22^. Both BRSK1 and BRSK2 phosphorylate microtubule-associated proteins (MAP)/Tau that regulate microtubule stability^23^. Furthermore, BRSKs act as a checkpoint kinase, because they phosphorylate Wee1 and cell division cycle 25 B and C (CDC25B/C) and regulate neuronal polarization^24^. NUAK2 is also called SNF1/AMPK-related kinase (SNARK) and this SNF1 is the yeast homologue of AMPK, which is a key regulator of cellular energy homeostasis and play a major role in switch/sucrose nonfermentable (SWI/SNF) chromatin remodeling complex. This SNF1/SWI complex contains either ATPase Brm or Brg1 and Bmi-1, and all of them play important role in neural development^18^,^25^ as well as HIV infection and disease progression^26^. Recent studies demonstrated that the Brg-1, a cyclin dependent kinase 5 (CDK5) implicate neuronal impairments including plasticity and memory dysfunction^27^. Also, the energy for SNF/SWI-mediated epigenetics remodeling is transduced by the catalytic subunit, Brm or Brg1, both of which have DNA-dependent ATPase activity^28^. In addition, this SNF/SWI complex is a CDK5 cell cycle protein and it may play a role in energy deficit leading to cell cycle arrest.

Overall, recent experimental evidence suggests that energy deficit lead to an important regulatory element in drug addiction and also in the development of neurodegenerative diseases^29^,^30^. Since glia are the major source of energy storage, they compensate the energy loss exporting lactate as an energy source that can be utilized by neurons and maintain cellular function and protective mechanisms^31^,^32^. Recent studies have demonstrated that cocaine affects glucose metabolism and subsequently impact various regions of the brain^33^. Clinical observations suggest that the cocaine abuser as well as patients with severe progression of HIV infection may present with energy consumption and metabolic deficits resulting in neuronal impairment. The role of energy sensor AMPKs mediated activation of BRSKs and SNARK proteins leading to metabolic deficit have not been studied yet with respect to the combine effect of cocaine and HIV infection. Furthermore, how HIV-1 and cocaine impact mitochondrial biogenesis in ATP utilization, oxygen consumption rate (OCR) and the secretion of extracellular acidification rate (ECAR) is not well understood. In the present study, we aimed to investigate the effect of HIV infection or HIV-1 gp120 protein with cocaine exposure in neuropathogenesis underlining energy deficits and metabolic dysfunction as important mechanism in the process. We highlight the role of energy sensor AMPKs and mitochondrial biogenesis with subsequent impact of epigenetic remodeling on the complex SWI/SNF which may have potential role in progression to neuroAIDS.

## Results

### Cocaine enhances HIV-1 gp120 induced OCR/ECAR

**Figure 1:**
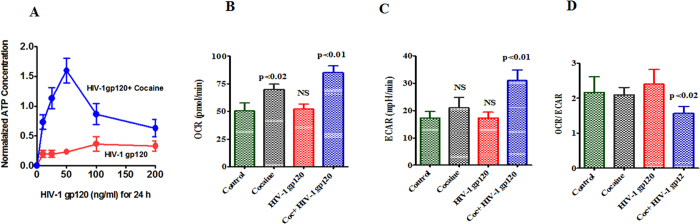
HIV-1 gp120 protein and cocaine exposure impact ATP utilization, oxygen consumption and extracellular acidification in microglia. CHME-5 (10,000 cells/ml) were treated with HIV-1 gp120 (0–100 ng), cocaine (0.5 μM) and combination of HIV-1 gp120 with cocaine for 24 h. Controls were maintained by drug free medium. At the end of the incubation period; the OCR and ECAR were measured by Seahorse Bioscience-XF96 extracellular flux analyzer. Data presented is the average of three independent experiments conducted under the same experimental conditions.

Studies have shown that HIV positive subjects who have used illicit drugs are more sensitive to neuropathogenesis than either substance abusers or HIV infection alone^5^,^6^,^34^. In this study, we have investigated the interaction of HIV-1 gp120 protein with cocaine exposure on the metabolic resources of OCR and ATP–utilization, and their subsequent interaction with energy sensor AMPKs. Furthermore, we studied these interactions in the downstream signaling intermediate in acetyl-coA carboxylase (ACC), CDC25B/C, MAP/Tau, Wee1 and oxidative damage of mitochondrial biogenesis, and SWI/SNF lead neuropathogenesis in CHME-5 cells. Data presented in Fig. 1 shows that effect of HIV-1 gp120 is significantly enhanced by cocaine when measured for ATP concentration (A), measurements of oxygen consumption (OCR) (B), extracellular acidification rates (ECAR) (C) and OCR/ECAR ratio (D) in CHME-5 cells. Figure 1A shows no changes in utilization of ATP by HIV-1 gp120 exposure whereas cocaine exposure with HIV-1 gp120 significantly upregulated ATP concentration. Figure 1B shows a significant increase in OCR in cocaine treated (p < 0.02) and cocaine co-treated with HIV-1 gp120 (p < 0.01) compared to control. Since altered OCR level might affect ATP utilization it may play a role in OXPHOS. Therefore, we examined whether ATP utilization affects metabolic ratio of OCR/ECAR. Figure 1D results indicate that cells exposed with cocaine and HIV-1 gp120 (p < 0.02) have a significantly lower OCR/ECAR ratio compared to control.

### HIV and cocaine interactive effect on energy sensor AMPKs and epigenetic remodeling complex SWI/SNF expression

**Figure 2:**
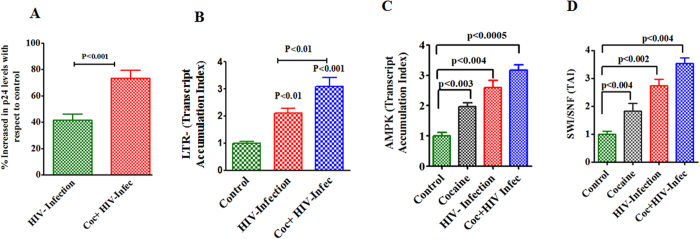
Effects of HIV infection and cocaine co-morbidity impact energy sensor AMPK and epigenetic remodeling complex SWI/SNF in microglia. CHME-5 (50 × 10^5 ^cells/ml) were infected with the HIV Bal strain TCID_50_ for 18 hours. After that, treated with cocaine (0.5 μM) at every 72 h and the supernatants were collected at 7^th^ day and used to estimate p24 antigen by ELISA (**A**) and infected cells total RNA was extracted and reverse transcribed followed by quantitative real time PCR for LTR (**B**), AMPK-α (**C**) and SWI/SNF-1 expression (**D**). Data are expressed as mean ± SE of TAI values of three independent experiments conducted under the same experimental conditions.

Figure 2 shows HIV infection is significantly accelerated by cocaine as monitored by measuring p24 levels (A), LTR (B), AMPK-α (C) and SWI/SNF (D) expression in CHME-5 cells. Data presented in Fig. 2A show that cocaine exposure with HIV infection have significantly increased p24 levels (p < 0.001) compared to HIV infection alone. The level of LTR in HIV-infected cells in the presence of cocaine is significantly higher than the HIV infected cells (Fig. 2B). Since increased HIV-LTR levels might results in modulation of intracellular AMPK expression and it may impact epigenetic function, we analyzed the AMPK-α and epigenetic remodeling complex SWI/SNF expression level during HIV infection and cocaine exposure. Figure. 2C shows AMPK-α is significantly upregulated by cocaine (p < 0.003), HIV-infection (p < 0.004) and cocaine exposure with HIV infection (p < 0.005). Figure. 2D represents the expression level of SWI/SNF in cocaine (p < 0.004), HIV-infection (p < 0.002) and cocaine with HIV infection (p < 0.004), respectively.

**Figure 3:**
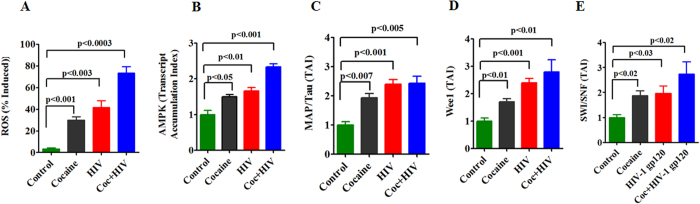
Effects of HIV-1 gp120 protein and cocaine co-morbidity induced oxidative stress altered energy sensor AMPK and epigenetic remodeling complex SWI/SNF in microglia. CHME-5 (1 × 10^6^ cells/ml) were treated with HIV-1 gp120 (50 ng), cocaine (0.5 μM) and combination of HIV-1 gp120 with cocaine for 24 h. Controls were maintained by drug free medium. At the end of the incubation, ROS production was analyzed by flow cytometry (**A**), the total RNA was extracted and reverse transcribed followed by quantitative real time PCR for AMPK-α (**B**), MAP/Tau (**C**), Wee1 (**D**) and SWI/SNF-1 (**E**) and housekeeping β-actin specific primers. Data are expressed as mean ± SE of TAI values of three independent experiments conducted under the same experimental conditions.

In addition, we have also studied HIV-1 gp120 protein with cocaine which resulted in the level of ROS production, AMPK-α, MAP/Tau, Wee1 and SWI/SNF gene expression. Figure 3A indicates that the level of ROS in HIV-1 gp120 with cocaine is significantly increased (P < 0.0003) when compared to either cocaine (p < 0.01) or HIV-1gp120 (P < 0.003) alone. Figure. 3B shows AMPK is significantly increased in cocaine (p < 0.05), HIV-infection (p < 0.01) and cocaine with HIV infection (p < 0.001). Figure. 3C shows MAP/Tau is also significantly upregulated due to cocaine (p < 0.007), HIV-infection (p < 0.001) and cocaine with HIV infection (p < 0.005). The transcriptional factor Wee1 and SWI/SNF expression are significantly upregulated during cocaine with HIV infection/gp120 protein when compared with control. Expression level of Wee1 during cocaine (p < 0.01), HIV-infection (p < 0.001), cocaine exposure with HIV infection (p < 0.01), SWI/SNF expression levels due to cocaine (p < 0.02), HIV-infection (p < 0.03) and cocaine exposure with HIV infection (p < 0.02) altered significantly. Overall results indicate that HIV infection or HIV-1 gp120 with cocaine significantly upregulated AMPKs, MAP/Tau, Wee1, and SWI/SNF expression when compared with HIV infection/gp120 protein or cocaine alone. These results suggest that HIV infection alters metabolic deficits accelerated by cocaine and it may increase viral replication and disease progression.

### Cocaine exacerbates HIV-1 gp120 induced protein activation

**Figure 4:**
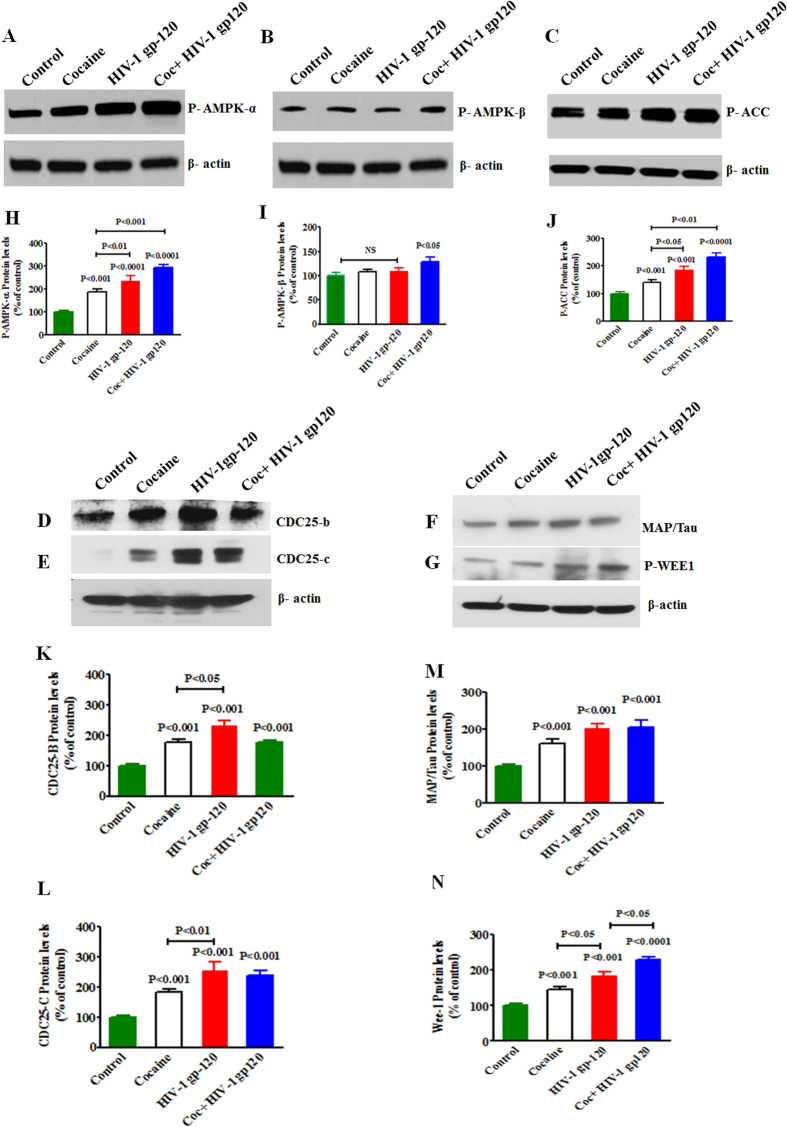
HIV-1 gp120 and cocaine impacts AMPK signaling network. CHME-5 (1 × 10^6^ cells/ml) were treated with HIV-1 gp120 (50 ng), cocaine (0.5 μM) and combination of HIV-1 gp120 with cocaine for 24 h. Controls were maintained by drug free medium. At the end of the incubation, equal amount of protein lysate were resolved by 4–15% SDS-PAGE and protein expression were analyzed by Western blot showing AMPK-α (**A**), AMPK-β (**B**), P-ACC (**C**), CDC25B (**D**), CDC25C (**E**), MAP/Tau (**F**) and WEE1 (**G**). **(H–N)** represent % densitometric values of protein levels (% control), respectively. Data are expressed as mean ± SE of three independent experiments conducted under the same experimental conditions.

In order to validate the increased gene expression by qRT-PCR, we also investigated their protein expression by western blot analysis (Fig. 4). The immune blots shows expression of AMPK-α (A), AMPK- β (B), P-ACC (C), CDC25B (D), CDC25C (E), MAP/Tau (F) and Wee1 (G) with the exposure of cocaine, HIV-1 gp120 and cocaine with HIV-1 gp120. The corresponding densitometry analysis was represented in Fig. 4 AMPK-α (H) (p < 0.0001), AMPK-β (I) (p < 0.05), ACC (J) (p < 0.0001), CDC25B (K) (p < 0.001), CDC25C (L) (p < 0.001), MAP/Tau (M) (p < 0.001), and Wee1 (N) (p < 0.0001) respectively.

### Cocaine and HIV-1 gp120 effect on BRSKs Protein

**Figure 5:**
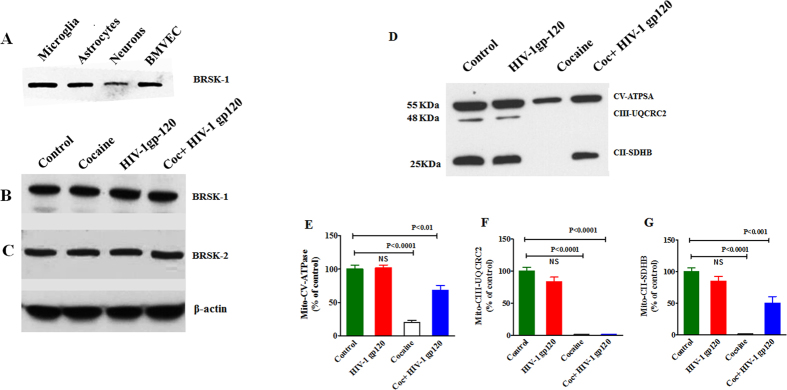
Effect on BRSKs and oxidative damage on mitochondrial proteins. CHME-5 (1 × 10^6 ^cells/ml) were treated with HIV-1 gp120 (50 ng), cocaine (0.5 μM) and combination of HIV-1 gp120 with cocaine for 24 h. Controls were maintained by drug free medium. At the end of the incubation, equal amount of protein lysate were resolved by 4–15% SDS-PAGE and protein expression were analyzed by Western blot showing neuronal cells level in BRSK1 (**A**), and CHEM-5 cells in BRSK 1 (**B**), BRSK-2 (**C**), and mitochondrial proteins (**D**). (**E**–**G**) represent % densitometric values of CV-ATPase, CIII-UQCRC2 and CII-SDHB protein levels (% control). Data are expressed as mean ± SE of three independent experiments conducted under the same experimental conditions.

Observed results demonstrated that there was a significant upregulation of AMPKs expression in cocaine with HIV infection/HIV-1 gp120 protein when compared to either cocaine or HIV infection/HIV-1 gp120 protein alone. Since BRSK(s) belong to AMPKs families and it may be affected by HIV and cocaine. It is important to measure BRSKs expression. Therefore, we have analyzed the effect of cocaine and HIV-1 gp120 protein on BRSKs expression which has two isoforms. Data presented in Fig. 5 shows BRSK1 expression level in CNS cells such as microglia, astrocyte, neuron and brain microvascular endothelial cells (BMVEC) (A). Figure. 5B,C demonstrate BRSK expression in CHME-5 microglial exposed to cocaine, HIV-1 gp120 and cocaine with HIV-1 gp120 compared with control. Interestingly, BRSK1 (B) and BRSK2 (C) expression levels are not affected by either cocaine or HIV-1 gp120 protein. These results suggest that, HIV or cocaine exposure disrupt AMPKs metabolic resources of energy consumption and utilization without affecting BRSKs proteins, which leads to dysregulation of downstream signals MAP/Tau and Wee1 transcriptional function.

### Cocaine and HIV-1 gp120 effect on mitochondrial biogenesis

Mitochondria plays a prominent role in production of energy source (ATP) of cells through respiration and maintenance of cellular function. Once mitochondria is altered, AMPKs expression subsequently affect OXPHOS. Mitochondria were isolated and analyzed by Western blot to determine whether AMPKs impact energy deficit and ATP utilization to induce mitochondrial oxidative damage. Figure. 5D shows that CHME-5 cells treated with HIV-1 gp120 had no effect on mitochondria whereas cocaine significantly inhibit OXPHOS and affect mitochondrial biogenesis. Interestingly, HIV-1 gp120 had no effect on mitochondrial protein but cocaine exposure (p < 0.0001) and cocaine with HIV-1 gp120 (p < 0.01) had significant effect on mitochondrial ATP synthase or complex V (CV-ATPase) (Fig. 5E). The ubiquinol–cytochrome *c* reductase core protein II (UQCRC2) of complex III (CIII-UQCRC2) (Fig. 5F) and succinate dehydrogenase (ubiquinone) iron–sulfur subunit (SDHB) of complex II (CII-SDHB) (Fig. 5G) significantly inhibits OXPHOS in cocaine with HIV-1 gp120 compared with either cocaine or HIV-1 gp120 alone. This suggest that inhibition of CIII-UQCRC2 and CII-SDHB affects mitochondrial respiration, which could enhance viral replication and disease progression.

### Cocaine and HIV-1 gp120 effect on epigenetics remodeling complex SWI/SNF

**Figure 6:**
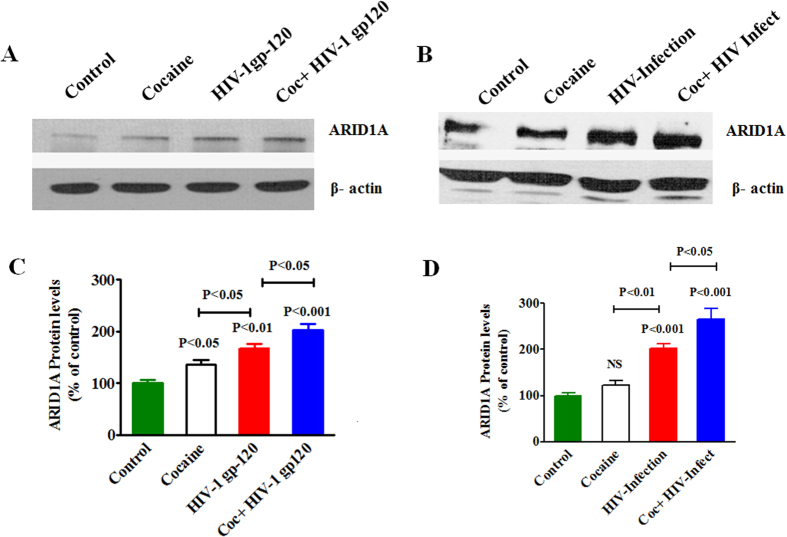
HIV infection and HIV-1 gp120 co-morbidity with cocaine effect on epigenetics remodeling protein. CHEM-5 (1 × 10^6^ cells/ml) were infected with the HIV Bal strain TCID_50_ for 18 hours. After that, treated with cocaine (0.5 μM) every 72 h. In another set of experiment, CHME-5 (1 × 10^6^ cells/ml) were treated with HIV-1 gp120 (50 ng), cocaine (0.5 μM) and combination of HIV-1 gp120 with cocaine for 24 h. At the end of the incubation, equal amount of protein lysate were resolved by 4–15% SDS-PAGE and protein expression were analyzed by Western blot showing HIV infected with cocaine effect on SWI/SNF protein ARID1A (**A**) and HIV-1 gp120 protein with cocaine (**B**). (**C**,**D**) represent % densitometric values protein levels (% control), respectively. Data are expressed as mean ± SE of three independent experiments conducted under the same experimental conditions.

Furthermore, the liver kinase B1 (LKB1)-AMPKs localize not only to the cytoplasm but also to the nucleus, suggesting a possible nuclear role for the interaction and activation of brahma-related gene-1 (BRG1), a catalytic subunit of SWI/SNF chromatin remodeling complex^35^. Therefore, to see any effect SWI/SNF complex protein, we have infected cells with HIV virus (Bal) or HIV-1 gp120 protein, in the presence or absence of cocaine exposure. Figure 6 showed SWI/SNF protein AT-rich interactive domain-containing protein (ARID1A) expression in HIV-1 gp120 with cocaine (Fig. 6A) and HIV infection with cocaine (Fig. 6B) which were significantly upregulated when compared with control. Figure 6(C,D) shows the densitometry analysis of ARID1A expression in the presence of HIV-1 gp120 protein and HIV infection indicating significant upregulation during cocaine (p < 0.05), HIV-1 gp120 (p < 0.01) and HIV-1 gp120 with cocaine (p < 0.001), and HIV infection (p < 0.01) and cocaine with HIV infection (p < 0.001). The observed results of energy sensor AMPKs proteins oxidative damage of mitochondrial biogenesis subsequently upregulate SWI/SNF complex in cocaine with HIV infection compared to either cocaine or HIV infection alone.

## Discussion

Epidemiological and clinical studies have demonstrated that illicit drugs accelerate viral replication and disease progression, which synergistically impair neuronal functions in HIV positive subjects^36^,^37^,^38^,^39^. Studies have also shown that cocaine exacerbates HIV infection implicating neuro-immune dysfunction in *in vitro* as well as HIV positive subjects^4^,^5^,^7^. Glial cells are the major resource of energy storage and maintenance of cellular homeostats in CNS. These metabolic functions in energy consumption and utilization are regulated by AMPKs^17^,^19^,^20^ and also involved in cell architecture, cell polarity and ion transport control^40^. However, activation of AMPKs lead to higher ROS, and subsequently increase AMP: ATP ratio, which might play a vital role in ATP depletion and mitochondrial damage leading to neuronal dysfunction^41^. It is known that microglia is the major reservoir of HIV infection and disease progression^1^,^2^; and these cells transfer energy to neurons. However, there are no reports on the effects of HIV infection and cocaine-induced metabolic dysfunction and energy impairment which affect energy sensor AMPKs, mitochondrial biogenesis and chromatin remodeling complex SWI/SNF. Interestingly, how HIV infection as well as cocaine exposure impact metabolic dysfunction in independent pathways, which do not affect BRSKs are need to be further studied. The observed results provide new insights into the functional role of AMPKs expression altered mitochondrial biogenesis which subsequently affect epigenetic remodeling complex SWI/SNF. HIV infection and cocaine interaction are known to induces oxidative stress and ROS production^7^,^42^, and it may activate energy sensor AMPKs, mitochondrial biogenesis and subsequently impact downstream signals for CDC25B, CDC25C, MAP/Tau, Wee1 and SWI/SNF transcription, which may play a vital role in neuronal dysfunction in HIV associated neurocognitive disorder (HAND).

In the present study, we have shown for the first time that cocaine with HIV infection or HIV-1 gp120 protein impact additively the of ATP utilization, ECR/OCAR ratio (Fig. 1), ROS production and activation of AMPKs, and CDC25B, CDC25C, MAP/Tau and Wee1 expression (Figs 2 and 3) associated with inhibition of mitochondrial oxidative phosphorylation. The increase in AMPKs expression subsequently upregulates Wee1 and SWI/SNF transcription, which may enhances viral replication and disease progression. These results suggest that cocaine accelerates HIV infection as well as HIV-1 gp120 protein may have an enhanced role on AMPKs and SWI/SNF complex when compared to control. This is consistent with earlier reports of HIV infection impacting AMP: ATP ratio and utilization of energy metabolites and leading to AMPKs and SWI/SNF pathways^43^,^44^. The BRSKs and SNARK proteins are closely associated with energy metabolic role^45^ and act as a checkpoint kinase, as it phosphorylate Wee1 and CDC25B/C, and regulates neuronal polarization^24^. Studies have also shown that HIV-1 gp120 and Vpr activate CDC25B/C mediated cell cycle arrest, which may lead to adult neurogenesis and dementia^46^. However, recently we have also shown that HIV-1 gp120 with morphine significantly accelerates the expression of CDC25B/C and tentatively impact cell cycle process^47^. These findings are consistent with our present observation on MAP/Tau and CDC25B/C which are involved in energy deficits leading to neuropathogenesis (Fig. 4).

Two other AMPK-related kinases, BRSKs proteins (BRSK1 and BRSK2), are also highly expressed in the mammalian brain and are crucial for neuronal development. We have also analyzed BRSKs expression in CNS cells (primary astrocytes and BMVEC, and SK-N-MC neuroblastomas) and these kinases are predominantly found in all these cells (Fig. 5). Interestingly, BRSKs (BRSK1 and BRSK2) are not affected by either HIV-1 gp120 or cocaine exposure in microglia (Fig. 5B,C). It is known that BRSKs play a wide role in CNS especially in neuronal polarity whereas no studies have reported yet in glial cells. In this present study we have tried to find out whether these kinases are involved in any metabolic network in microglia. Surprisingly, these proteins are not involved in either HIV or cocaine impact on metabolic ratio suggesting that it may work on an independent pathway regulating without affecting BRSKs. Studies have also shown that AMPK and BRSK regulate neuronal polarization and vesicular transmission in neuron^22^. Interestingly, AMPKs without affecting BRSKs induce neurodegeneration which will be a novel pathway and its functions are reaming unknown.

Besides, oxidative stress and ROS production inhibit redox expression and affect mitochondrial biogenesis and oxidative phosphorylation which are known to regulate post-translational modifications induced by the energy sensors, AMPK^48^. Also, mitochondrial dysfunction is associated with the pathogenesis of several neurodegenerative diseases, including AD^49^ and PD^50^. Studies have shown that mitochondrial dysfunction plays a critical role in the pathologic mechanisms of neurologic disorders and associated with the drugs of abuse and HIV infection^51^,^52^. Dysfunctional mitochondrial machinery induces the disruption of energy metabolism that culminates with a decrease in ATP production, Ca2+ buffering impairment and exacerbated generation of ROS^53^. Whereas brain mitochondrial dysfunction is an important component of neurodegeneration, and significant disruption of mitochondrial respiration^49^,^54^. These observed results suggest that increased ATP utilization subsequently shutdown the energy consumption pathway and inhibit mitochondrial OXPHOS which could possibly be at least partially responsible for the energy dysfunction effects (Fig. 5D,E). Studies have also shown that cocaine induced oxidative stress impact brain mitochondrial function and energy metabolism in cocaine abusers^55^, and HIV positives^56^. Defective OXPHOS may be caused by abnormal mitochondrial biosynthesis due to inherited or acquired mutations in the nuclear (n) or mitochondrial (mt) deoxyribonucleic acid (DNA). It is know that OXPHOS inhibiting CV-ATPase and CIII-UQCRC2, and CII-SDHB play a vital role in induction of mitochondrial DNA.

Epigenetics remodeling complex SWI/SNF is a CDK5, a cell cycle protein and it may play role in energy deficits leading to cell cycle arrest. However, the mammalian SWI/SNF chromatin-remodeling complex plays an essential roles in a variety of cellular processes including differentiation, proliferation and DNA repair, which are altered by mitochondrial biogenesis. These functions are ATP-dependent, which use energy from ATP hydrolysis to disrupt energy production and utilization interactions, containing one of two catalytic ATPase subunits, called *Brm* and *Brg1*. Both Brg1 and Brm have been implicated in transcriptional activation and repression^28^. Several SWI/SNF-related factors have been implicated in transcriptional silencing through cell cycle arrest^22^. These studies further confirm that the downstream cascade CDC25B/C, wee1, and SNF/SWI are upregulated in cocaine along with HIV-1 gp120 exposure leading to cell cycle arrest. However, CDC25B/C are direct targets of CDK5^57^, which may be mediated by CDC25B/C leading to neuronal impairment. Supporting our hypothesis, recent studies have demonstrated that the Brg-1, a CDK5, results on neuronal impairments including plasticity and memory dysfunction^27^,^58^. These results confirm that increased AMP: ATP utilization and activation of AMPKs exacerbate the viral replication and disease progression in cocaine use during HIV infection.

**Figure 7:**
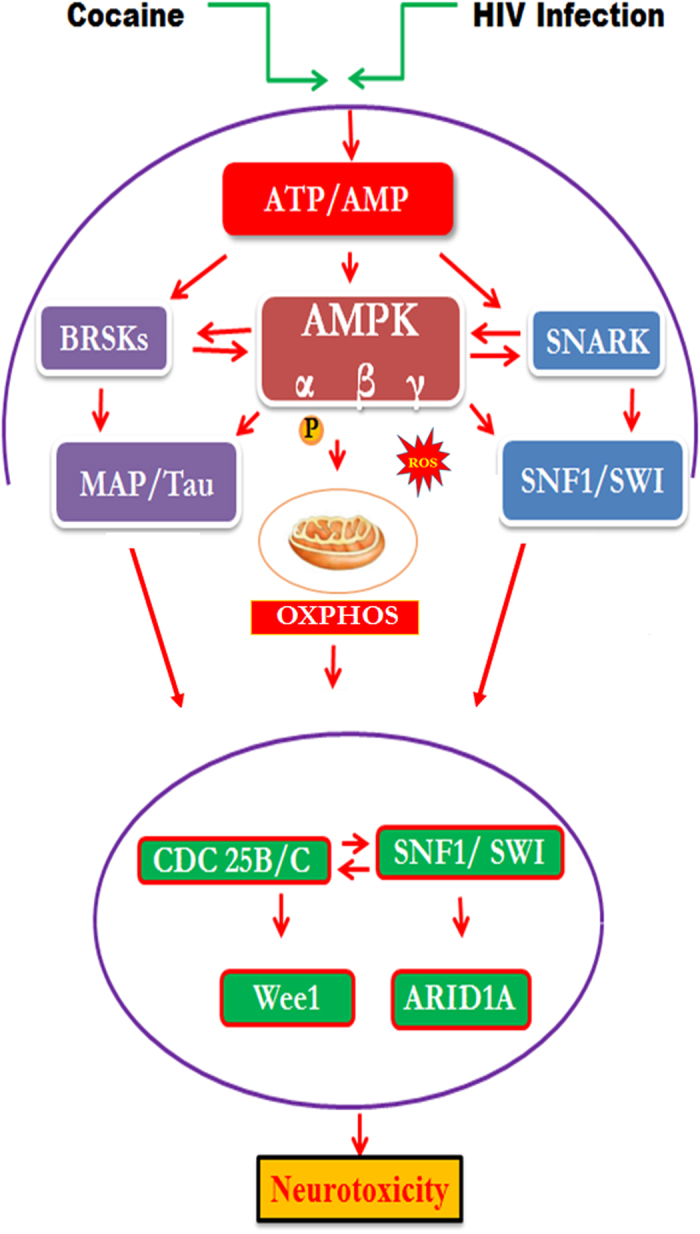
Schematic pathway of HIV infection and cocaine impact on microglial energy resource and influence neuronal toxicity. A comprehensive model showing how HIV-gp120 and cocaine effect energy sensor AMPKs signaling mechanism and epigenetic signature lead neurotoxicity. HIV infection and HIV envelop protein (gp120) leads to altered AMP/ATP ratio and AMPKs activations’ which influence mitochondrial biogenesis and subsequent induction of MAP/Tau and CDC25B/C mediated epigenetic remodeling SWI/SNF complex, and that may lead neurotoxicity.

Further, our results show that cocaine treatment, HIV infection, and HIV-1 gp120 protein cause an induction of SWI/SNF protein ARID1A expression (Fig. 6). ARID1A is a member of the SWI/SNF family and is a unique component of the BRG1, whose members have helicase and ATPase activities and are thought to regulate transcription of certain genes by altering the chromatin structure around those genes. The main observation in this report is that HIV infection or HIV-1gp120 protein with cocaine have upregulated AMPKs activation and subsequently inhibit mitochondrial OXPHOS, which leads to increase in the expression level of MAP/Tau and SWI/SNF protein ARID1A (Fig. 7). However, HIV-1 along with cocaine exposure has significantly upregulated SWI/SNF complex and ARID1A protein which is associated with a concomitant activation of ATP utilization and AMPKs activation. Studies have shown that contractual report on ARID1A expression, however, over expression of ARID1A impairs liver proliferation and regeneration^59^. These results suggest that there is an interactive role between cocaine and HIV that additively potentiate and increase co-morbidity when compared to either cocaine or HIV infection alone.

Overall, the data provides evidence in the interaction of cocaine with HIV infection and gp120 protein induction which is associated with activation of AMPKs and subsequently affects energy sources. Based on these results, we have demonstrated that energy deficits potentiate HIV viral replication and disease progression by impairing the glial function, which may lead to neurodegeneration. The present study support some of earlier reports in the dysfunction of energy resources^60^ and increased neurodegeneration in cocaine using HIV-1 Tat protein^61^,^62^. These observations lead to demonstrate that energy sensor AMPKs, mitochondrial biogenesis and chromatin remodeling complex SWI/SNF plays a major role and contributes in viral replication and disease progression in cocaine abusers. These findings provide a novel pathway to explain neurological deficits in neuropathogenesis associated with cocaine exposure during HIV infection.

## Methods

### HIV virus and gp120 proteins

The HIV-1 Bal strain and recombinant gp120 (Bal) proteins were obtained from the NIH AIDS Research and Reference Reagent Program. The recombinant HIV-1 gp120 proteins were >95% purity.

### Cell cultures

In this study, we used immortalized CHME-5 microglial cells, primary astrocytes, BMVC and SK-N-MC neuroblastoma cell lines. Primary cells astrocytes and BMVC were maintained in basal medium containing 10% fetal bovine serum, 50 units/ml of penicillin, with growth supplement, and 100 μg/ml of streptomycin (Sciencell, Carlsbad,CA). The neuroblastoma cell line, SK-N-MC was purchased from ATCC (catalog # HTB-10;Manassas, VA) and the immortalized CHME-5 microglial cells were cultured in Eagle’s minimum essential medium (MEM) supplemented with fetal bovine serum to a final concentration of 10% and 1% antibiotic ⁄ antimycotic solution (Sigma-Aldrich, St. Louis, MO).

### Measurements of oxygen consumption and extracellular acidification

Oxygen consumption rate (OCR) and extracellular acidification rate (ECAR) was measured using XF96 extracellular flux analyzer (Seahorse Bioscience, North Billerica, MA)^63^. CHME-5 (10,000 cells/well) were incubated at 37 °C/10% CO_2_ incubator for 24 h in 96 cell culture plate. After 24 h, growth medium was exchanged with XF assay medium (DMEM supplemented with 25 mM glucose, 4 mM L-glutamine and 1 mM pyruvate with low phosphate and without bicarbonate). Cells were treated with increasing concentration of HIV-1 gp120 protein (0–100 ng/ml) with or without cocaine (0.5 μM) at 24 hr. End of the incubation period; the OCR and ECAR were measured and expressed as percentage.

### HIV virus infection and gp120 protein treatment

CHME-5 (50 × 10^5 ^cells/ml) were treated with polybrene (4 μg/ml) for 5 hours and then infected with HIV-1 Bal at 20 ng/10^6^ or TCID_50_ at 37 °C. After 18 hours, the cells were washed with PBS, and the HIV infections were maintained for 7 days as previously described^64^. CHME-5 (1 × 10^6^ cells/ml) was treated with HIV-1 gp120 protein (50 ng/ml) for 24 h. The untreated cells served as the negative control.

### RNA extraction and real-time quantitative PCR (qRT-PCR)

Total RNA from CHME-5 microglial cells was extracted using a Qiagen kit (Invitrogen Life Technologies, CA, USA) followed by cDNA synthesis using the high capacity reverse transcriptase cDNA kit (Applied Biosystems, Foster City, CA) to perform qRT-PCR using Taqman gene expression assays (Applied Biosystems, Foster City, CA) for LTR, AMPKs (α and β), MAP/Tau, Wee1 and SWI/SNF were normalized to β-actin housekeeping gene^65^. The relative abundance of each mRNA species was assessed using the brilliant Q-PCR master mix from Applied Biosystems and the Stratagene Mx3000P instrument, which detects and plots the increase in fluorescence versus the PCR cycle number to produce a continuous measure of PCR amplification. Relative expression was quantitated for each mRNA species, and the mean fold change in the expression of the target gene was calculated using the comparative CT method (Transcript Accumulation Index, TAI = 2^−ΔΔCT^). All data were controlled for the quantity of RNA input by performing measurements on the endogenous reference gene β-actin. In addition, RNA results from treated samples were normalized to results obtained using RNA from the control, untreated sample.

Mitochondria Isolation. Mitochondrial Isolation Kit (Abcam, CA) was used to isolate mitrochondria from the treated cells Briefly, CHME-5 (3 × 10^8^) cells were grown and treated with HIV-1 gp120 (50 ng/ml) and cocaine (0.5 μM) at 24 h. End of the incubation, cells were split by 0.05% trypsin-EDTA and washed with PBS. The cells were pelleted by centrifugation (1000 × ***g*** for 15 min) at room temperature and suspended in mitochondrial assay buffer. Cells were incubated in the solution on ice for 2 min. CHME-5 cells were homogenized with a glass homogenizer using 10 up-down strokes, and cellular disruption was confirmed by microscopy. That supernatant was centrifuged at 5,000 × ***g*** for 10 min at 4 °C according manufactures protocol. The pellet was then resuspended in lysis buffer (Pierce, Rockford, IL) for Western blot analysis.

### Western blot analysis

To assess modifications of AMPKs, ACC, (Cell signaling, CA), BRSKs, CDC25B, CDC25C, MAP/Tau, Wee1 (Santa Cruz Biotechnologies, CA) and SWI/SNF protein ARID1A (Novus Biological Lab, Littleton, CO) in CHME-5 by treating with HIV-1 gp120 alone or in combination with cocaine, the cells were lysed using lysis buffer (Pierce, Rockford, IL, USA) with protease inhibitor cocktail. Equal amounts of total cellular proteins were resolved using 4–15% gradient polyacrylamide gel electrophoresis, transferred to a nitrocellulose membrane and incubated with respective primary antibodies (1:1000) for overnight and washed with TBST buffer and incubated respective secondary antibodies. Immunoreactive bands were visualized using a chemiluminescence western blotting system according to the manufacturer instructions (Amersham).

### Determination of Reactive Oxygen Species (ROS) production

The HIV-1 gp120 and cocaine induced ROS production were analyzed using flow cytometry in FACS caliber (BD Bioscience, San Jose, CA). Briefly, CHME-5 cells (5 × 10^5^) were treated HIV-1 gp120 with or without cocaine for 24 h. At the end of the time period, 2,7-dichlorodihydrofluorescein diacetate (10 μM) (DCFH-DA) (Invitrogen, Carlsbed, CA) was added to the medium and incubated at 37 °C for 30 min followed by washing with PBS then ROS production levels were analyzed.

### ELISA - p24 measurements

A commercially available enzyme linked immunosorbent assay (ELISA) kit (Zeptometrix, Buffalo, NY, USA) was used to quantitate p24 in culture supernatants.

### Data analysis

Results presented in this study are representative of three or more independent experiments performed in triplicate. Statistical significance was analyzed using Graph Pad Prism5 software, La Jolla, CA by performing ANOVA or the Student’s *t*-test for unpaired observations. The values are presented as means ± SEM, and *P* < 0.05 was considered to be significant.

## Additional Information

**How to cite this article**: Samikkannu, T. *et al*. HIV and Cocaine Impact Glial Metabolism: Energy Sensor AMP-activated protein kinase Role in Mitochondrial Biogenesis and Epigenetic Remodeling. *Sci. Rep.* **6**, 31784; doi: 10.1038/srep31784 (2016).
